# *PLAG1* fusions define a third subtype of CNS embryonal tumor with PLAG family gene alteration

**DOI:** 10.1007/s00401-025-02917-z

**Published:** 2025-08-02

**Authors:** Michaela-Kristina Keck, Maysa Al-Hussaini, Nisreen Amayiri, Akosua Adoma Boakye Yiadom, Gabriel Chamyan, Edmund Cheesman, Cécile Faure-Conter, Miguel Garcia-Ariza, Guillaume Gauchotte, Martin Hasselblatt, Mette Jorgensen, John-Paul Kilday, Gabriela Lamas, Cinzia Lavarino, Marilyn M. Li, Fabiana Lubieniecki, Ossama M. Maher, David Meyronet, Jan Mueller, Mariarita Santi, Ulrich Schüller, Ana Luiza Seidinger, Martin Sill, Sniya Sudhakar, María Tallón García, Arnault Tauziede-Espariat, Pascale Varlet, Alexandre Vasiljevic, Andrea Wittmann, Andreas von Deimling, David A. Solomon, Felix Sahm, Anna Tietze, Katja von Hoff, Philipp Sievers, David T. W. Jones

**Affiliations:** 1 Division of Pediatric Glioma Research, Hopp Children’s Cancer Center Heidelberg (KiTZ), Heidelberg, Germany; 2 National Center for Tumor Diseases (NCT), NCT Heidelberg, a partnership between DKFZ and Heidelberg University Hospital, Heidelberg, Germany; 3 German Cancer Research Center (DKFZ), Im Neuenheimer Feld 280, 69120 Heidelberg, Germany; 4 Department of Cell Therapy and Applied Genomics, King Hussein Cancer Center, Amman, Jordan; 5 Department of Pediatric Oncology, Neuro-Oncology Service, King Hussein Cancer Center, Amman, Jordan; 6 Department of Pediatric Hematology and Oncology, Nicklaus Children’s Hospital, Miami, FL USA; 7 Department of Paediatric Histopathology, Royal Manchester Children’s Hospital, Manchester, UK; 8 Institut d’Hemato-oncologie Pediatrique, Lyon, France; 9 Pediatric Hematology and Oncology Unit, Department of Pediatrics, Biobizkaia Health Research Institute, Hospital Universitario Cruces, Barakaldo, Spain; 10 Department of Biopathology, CHRU-ICL, Tumorothèque BB-0033-00035, INSERM U1256 NGERE, Vandoeuvre-lès-Nancy, France; 11 Institute of Neuropathology, University Hospital Münster, Münster, Germany; 12 Oncology Department Great Ormond Street Hospital for Children, NHS Foundation Trust, London, UK; 13 The Centre for Paediatric, Teenage and Young Adult Cancer, Division of Cancer Sciences, The University of Manchester, Manchester, UK; 14 Children’s Brain Tumour Research Network (CBTRN), Royal Manchester Children’s Hospital, Manchester University NHS Foundation Trust, Manchester, UK; 15 Department of Pathology, Hospital Nacional de Pediatria Dr JP Garrahan, Buenos Aires, Argentina; 16 Laboratory of Molecular Oncology, Pediatric Cancer Center Barcelona, Hospital Sant Joan de Déu, 08950 Barcelona, Spain; 17 Department of Pathology and Laboratory Medicine, The Children’s Hospital of Philadelphia, Philadelphia, PA USA; 18 Department of Pathology and Laboratory Medicine, The University of Pennsylvania Perelman School of Medicine, Philadelphia, Pennsylvania USA; 19 Institut de Pathologie Multisite-Site Est, Groupement Hospitalier Est, Hospices Civils de Lyon, Lyon, France; 20 Institute of Neuropathology, University Medical Center Hamburg-Eppendorf, Hamburg, Germany; 21 Department of Pediatric Hematology and Oncology, University Medical Center Hamburg-Eppendorf, Hamburg, Germany; 22 Research Institute Children’s Cancer Center Hamburg, Hamburg, Germany; 23 Boldrini Children’s Center, Laboratory of Molecular Biology, Campinas, Brazil; 24 Department of Neuroradiology, Great Ormond Street Hospital for Children, London, UK; 25 Department of Paediatric Hematology and Oncology, Álvaro Cunqueiro Hospital, Galicia Sur Research Foundation, Vigo, Spain; 26 Department of Neuropathology, GHU Paris - Psychiatry and Neuroscience, Sainte-Anne Hospital, Paris, France; 27 Université de Paris, UMR S1266, INSERM, IMA-BRAIN, Institute of Psychiatry and Neurosciences of Paris, Paris, France; 28 Department of Neuropathology, Institute of Pathology, University Hospital Heidelberg, Heidelberg, Germany; 29 Clinical Cooperation Unit Neuropathology, German Consortium for Translational Cancer Research (DKTK), German Cancer Research Center (DKFZ), Im Neuenheimer Feld 224, 69120 Heidelberg, Germany; 30 Department of Pathology, Stanford University School of Medicine, Palo Alto, CA USA; 31 Hopp Children’s Cancer Center Heidelberg (KiTZ), Heidelberg, Germany; 32 Institute of Neuroradiology, Charité-Universitätsmedizin Berlin, Freie Universität Berlin and Humboldt-Universität zu Berlin, Berlin, Germany; 33 Department of Pediatric Oncology and Hematology, Charité-Universitätsmedizin Berlin, Corporate Member of Freie Universität Berlin, Humboldt-Universität zu Berlin, and Berlin Institute of Health, Berlin, Germany; 34 Department of Paediatric and Adolescent Medicine, Aarhus University Hospital, Aarhus, Denmark

**Keywords:** Embryonal CNS tumor, *PLAG1*, *PLAGL1*, *PLAGL2*, *PLAG1* fusion

## Abstract

CNS embryonal tumors with PLAGL amplification (ET, PLAGL) are a recently described tumor type marked by amplification of one of the PLAG family genes, *PLAGL1* or *PLAGL2*. Separately, a supratentorial, ependymoma-like CNS tumor type with PLAG family alteration, namely *PLAGL1* fusion, was also reported (NET_PLAGL1). Here, we use DNA methylation profiling in combination with copy number, RNA-seq, and histological analysis to characterize and classify a novel group of CNS embryonal tumors harboring *PLAG1* gene fusions (n=12). Through our screening, we identified a subset of CNS tumors (n=12) epigenetically distinct from other known CNS tumor types, but clustering close to the *PLAGL1*- and *PLAGL2*-amplified ET, PLAGL subtypes in our t-SNE analysis. Copy number profiles indicated putative *PLAG1* fusions, which were confirmed in 9/12 tumors (not determined in 3/12). Different 5’ fusion partners (*ASAP1*, *ADGRG1*, *TMEM68*, *TCF4*, *CHD7*, *NCALD*, *HNRNPK*, *LOC105378102*) were identified that upregulate wild-type *PLAG1* through promoter hijacking. Expression analysis shows upregulation of *PLAG1* as well as *IGF2*, *DLK1*, *Desmin*, *CYP2W1*, and *RET,* which are also robustly expressed in *PLAGL1/2*-amplified tumors. Patient characteristics, survival data, and clinical/imaging analysis show additional similarities to *PLAGL1/2*-amplified tumors. Median age at diagnosis was 5 years, tumors were located throughout the neuroaxis, and original histological diagnoses were heterogeneous. The tumors demonstrated morphologic heterogeneity, with most composed of densely cellular areas of primitive small blue cells, alongside focal regions showing clear cell morphology, microcystic changes, and ependymoma-like perivascular pseudorosettes. Applied treatment regimens were also heterogeneous, but some favorable responses to therapy were observed. In summary, we describe a third subtype of PLAG family-altered pediatric CNS embryonal tumor characterized by *PLAG1* gene fusion, which leads to upregulation of *PLAG1* and downstream genes. We therefore propose to rename ET, PLAGL to ET, PLAG (CNS embryonal tumor with PLAG family gene alteration) together with a specification of the respective subtype.

**Supplementary Information:**

The online version contains supplementary material available at 10.1007/s00401-025-02917-z.

## Introduction

The current 2021 edition of the World Health Organization (WHO) Classification of Central Nervous System (CNS) Tumors presents for the first time a classification which is driven by integrating molecular features on top of classical histopathology [14]. It thus recognizes the importance of including molecular analyses, such as next-generation sequencing (NGS) and DNA methylation-based classification, in addition to morphology and immunohistochemistry (IHC) [4, 17]. This is an important paradigm shift, especially with regards to tumor types that share histological and morphological features, but differ molecularly, and can only be reliably discriminated and classified based on molecular analyses—such as the former group of primitive neuroectodermal tumors (PNET), or distinct ependymoma or medulloblastoma subtypes [3, 21, 24]. Furthermore, through the application of DNA methylation profiling, multiple new tumor types have been identified and characterized—including our recent publications on supratentorial neuroepithelial tumor with *PLAGL1* fusion (NET_PLAGL1), neuroepithelial tumor with *PATZ1* fusion (NET-PATZ1), CNS embryonal tumor with PLAGL amplification (ET, PLAGL) [2, 11, 22]—with potential to be added in the next edition of the WHO Classification.

A further novel and important aspect of the current WHO Classification is that it now clearly distinguishes between pediatric-type and adult-type CNS tumors that differ fundamentally from each other, by not only acknowledging differences in the CNS edition, but also dedicating a separate volume to pediatric tumors alone [17]. CNS tumors are the second most common tumor type in children age 0–14 years and the leading cause of cancer-related death in this age group, with gliomas being the most common CNS malignancy (~50%) and embryonal tumors constituting about 12.2% of all CNS neoplasms [18]. Excluding the relatively more frequent medulloblastomas and atypical teratoid/rhabdoid tumors (ATRTs), other embryonal tumors are rare and comprise around 1.8% of all CNS neoplasms in children age 0–14 years.

The above-mentioned ET, PLAGL, which was previously described by our group, is a rare entity presenting as a primitive, embryonal-like neoplasm, with lack of expression of typical differentiation markers, while concurrently expressing early neural lineage genes. In addition, these tumors show dysregulation of imprinted genes and overexpression of the candidate drug targets *RET* and *CYP2W1* [11]. ET, PLAGL can be subdivided into two epigenetically different subtypes that are marked by amplification and subsequent overexpression of either *PLAGL1* (6q24.2) or *PLAGL2* (20q11.21), and that show differences regarding their typical age of onset and clinical behavior [10]. A recently published case report presented another PLAG family gene alteration in the context of CNS tumors, namely one child and one adult with CNS tumors harboring gene fusions of *PLAG1* [26]. However, to date, it remains unclear, if these represent a novel, separate tumor type such as the *PLAGL1*-fused ependymoma-like tumors (NET_PLAGL1), or if they might be related to the two known tumor subtypes of ET, PLAGL.

Here, we applied genome-wide DNA methylation and copy-number profiling, targeted next-generation DNA sequencing, and RNA sequencing to further biologically characterize and assess whether CNS tumors with *PLAG1* gene fusions represent a third subtype related to the previously described ET, PLAGL. We identify promoter hijacking events with diverse fusion partners leading to upregulation of wild-type *PLAG1* (located on chromosome 8q12.1). *PLAG1*-fused tumors show major similarities in gene expression compared to *PLAGL1*- and *PLAGL2*-amplified tumors including upregulation of imprinted genes as well as *RET* and *CYP2W1*. Clinical presentation seems to most closely resemble *PLAGL2*-amplified tumors, though more data will be required to draw reliable conclusions about adequate treatment regimens.

To reflect the molecular details and clinical differences of each subtype and to include all three subtypes into the best applicable taxonomy, we propose to rename the previous CNS embryonal tumor with PLAGL amplification (ET, PLAGL) to CNS embryonal tumor with PLAG family gene alteration (ET, PLAG)—along with the specification of the respective subtype—*PLAG1*-fused, *PLAGL1*-amplified, or *PLAGL2*-amplified.

## Materials and methods

### Extraction of DNA/RNA

Nucleic acids were extracted using the automated Maxwell nucleic acid purification platform (Promega, Madison, WI, USA). RNA was extracted from fresh-frozen or formalin-fixed paraffin-embedded (FFPE) tissue samples with the Maxwell RSC simply RNA Tissue kit or the Maxwell 16 LEV RNA FFPE Kit, respectively. DNA was extracted from fresh-frozen or FFPE tissue samples with the Maxwell RSC Tissue DNA kit or the Maxwell RSC DNA FFPE kit, respectively, according to the manufacturer’s instructions. Samples not processed in Heidelberg were extracted according to the respective standard local procedures with corresponding QC measures.

### Genome-wide DNA methylation profiling

DNA from fresh-frozen or FFPE tissue samples was subjected to genome-wide DNA methylation profiling and was either processed at the Department of Neuropathology of the University Hospital Heidelberg, or at the DKFZ Genomics and Proteomics Core Facility, or at the respective collaborating institution using the Infinium Methylation EPIC (EPIC) BeadChip or Infinium Human Methylation 450k Bead Chip arrays (Illumina) according to the manufacturer’s instructions. A portion of the cases was acquired through uploads to www.molecularneuropathology.org. Methylation array processing, t-Distributed Stochastic Neighbor Embedding (t-SNE) dimensionality reduction, and copy number variation (CNV) analysis based on the raw intensities of the methylation array probes were performed as described previously [4, 13]. The Integrative Genomics Viewer (IGV) was used to visualize copy number variants (CNVs) of the respective amplified PLAG family genes [19]. IDAT files of the 12 *PLAG1*-fused cases are accessible through The German Human Genome-Phenome Archive (GHGA accession # GHGAS82289365639281).

### Hierarchical clustering of samples

The raw intensities of the methylation array probes were analyzed as described previously [4, 13]. The 10,000 most variable CpG sites were selected based on variance across all samples to reduce dimensionality while retaining the most informative features. Manhattan distances were calculated between samples using these selected features. Hierarchical clustering was performed using the Ward.D2 linkage method. The resulting dendrogram was visualized with samples colored according to the same color scheme used for the t-SNE visualization. Hierarchical clustering analysis was performed in R (v4.0.5) using the cluster and dendextend packages.

### Patients and samples

Tumor samples and retrospective clinical information were obtained through the Heidelberg University Department of Neuropathology, the German Cancer Research Center (DKFZ), or directly collected from the respective collaborating national or international institution in compliance with local regulations. This study was approved by the Ethics Committee of the University of Heidelberg (approval S-318/2022). Due to the retrospective design of this study, specific informed consent was waived, and all patient data were pseudonymized prior to analysis. The pre- and post-treatment imaging data were provided either directly by the respective local participating center or by the national radiology reference center, according to the patient/parental consent and local ethics approval. Further clinical data and serial MRI data were requested for all nine patients with confirmed *PLAG1* fusions (fusions not determined in 3/12), but could only be obtained for a subset of five patients. These five patients with confirmed gene fusion, complete clinical information and available MRI scans were included in the combined imaging and clinical analysis. A comprehensive summary of survival times, treatments, and outcomes (Figure 5c) was created using R version 4.4.2 [27]. More detailed information on the respective treatment regimens (drugs/radiation) is summarized in supplementary Tables S2 and S5. Tumor tissue for confirmation of the fusion and/or complete clinical and MRI data were only partly available for a further five cases classified as ET, PLAG, and *PLAG1*-fused. The available information is summarized in Table 1. No material or information (besides sex, *n* = 2, and age, *n* = 1) was available for two cases. A graphical patient summary (Figure 5a, b) was created using GraphPad Prism version 10.2.1 for Windows (GraphPad Software, Boston, Massachusetts, USA, www.graphpad.com) and a general overview over the available patient information is provided in supplementary Table S1.

**Table 1:** CNS embryonal tumors with *PLAG1* gene fusion lacking complete clinical documentation and/or unavailable MRI scans

ID	Fusion	Age group [years]	Treatment	Relapse	Relapse treatment	OS [years]	Status at last follow-up	Not available
A400	*LOC105378102::PLAG1* (outside report)	8-11	GTR, local RT (proton)	Relapse after 10 months	Surgery, RT (CSI/boost), Avastin	1.8	DOD	MRI
A139	*TCF4::PLAG1* (outside report)	4-7	GTR, RT (whole-brain, CSI)	Local and metastatic relapses starting after 4 months	GTR, CT, CSI	3.6	alive	Dosage of RT, MRI
A403	ND	8-11	GTR, CT, RT	No relapse, no known metastasis	NA	20.8	alive	Type of CT, type and dosage of RT, MRI
A405	*NCALD::PLAG1*	0-3	STR	No known metastasis, progression after 15 months	NA	1.5	alive	Treatment data progression, MRI
A137	*HNRNPK::PLAG1*	8-11	STR, CT, RT	Metastasis at presentation, relapse after 8 months	GTR, CT	7.9	alive	Type of CT, type and dosage of RT, MRI

Listed are the five cases that dropped out of the clinical analysis due to incomplete clinical documentation or unavailability of MRI scans

### MRI analysis

MRI analysis was performed by an experienced pediatric neuroradiologist (AT) employing well-established MRI criteria [28] as described in Tietze et al. [29]. The evaluation of response adhered to the guidelines of the European Society for Pediatric Oncology (SIOPE) Brain Tumour Group [16].

### RNA sequencing and analysis

RNA used for the detection of gene fusions through RNA sequencing was either derived from fresh-frozen or FFPE tissues. RNA used for quantitative gene expression analysis was derived from fresh-frozen tissues. Quality of input RNA was assessed using the Agilent Bioanalyzer or TapeStation and sequencing was performed at the Neuropathology Department of the UKHD on an Illumina NovaSeq 6000 instrument as previously described (FFPE) [23], at the DKFZ Genomics and Proteomics Core Facility using an Illumina HiSeq4000 or NovaSeq device (fresh-frozen-Illumina TruSeq strand-specific PolyA+ libraries), or at the collaborating institutions. Fastq files from transcriptome sequencing were used for de novo annotation of fusion transcripts using the deFuse [15] and Arriba (v1.2.0) [30] algorithms with standard parameters. Gene expression analysis was performed with the R2 Genomics Analysis and Visualization Platform (http://r2.amc.nl) using a reference cohort of embryonal tumors (*n* = 116), glial tumors (*n* = 142), and normal fetal and adult brain tissues (*n* = 36) that had been processed the same way.

### Histology and immunohistochemistry

Histopathological review was retrospectively performed on a subset of the tumors (*n* = 4). Hematoxylin and eosin (H&E) and immunohistochemical staining was performed at the Department of Neuropathology of the University Hospital Heidelberg. Immunohistochemistry was performed on FFPE tissue sections on a Ventana BenchMark ULTRA Immunostainer using the ultraView Universal DAB Detection Kit (Ventana Medical Systems, Tucson, AZ, USA) and the following antibodies: glial fibrillary acid protein (GFAP; Z0334, rabbit polyclonal, 1:1000 dilution, Dako Agilent, Santa Clara, CA, USA), oligodendrocyte lineage transcription factor 2 (OLIG2; clone EPR2673, rabbit monoclonal, 1:50 dilution, Abcam, Cambridge, UK), Synaptophysin (clone MRQ-40, rabbit monoclonal, 1:160 dilution, Cell Marque Corp., Rocklin, CA, USA), epithelial membrane antigen (EMA, GP1.4, mouse monoclonal, dilution 1:1000, Thermo Fisher Scientific), Ki-67 (clone MIB-1, mouse monoclonal, dilution 1:100, Dako Agilent), LIN28 (A177, rabbit polyclonal, 1:50, Cell Signaling, Danvers, MA, USA), S100 (Z0311, rabbit polyclonal, 1:2000, Dako Agilent), Desmin (clone D33, mouse monoclonal, dilution 1:50, Dako Agilent), and CD99 (CONFIRM anti-CD99, O13, mouse, monoclonal, Roche, Basel, Switzerland). Representative H&E-stained sections and immunohistochemical stains were digitally scanned on an Aperio slide scanner and histological and immunohistochemical features were reviewed and annotated using ImageScope software (Leica Biosystems).

### Targeted next-generation DNA sequencing

Genomic DNA extracted from FFPE tumor tissue was used for targeted next-generation DNA sequencing (NGS) at the UCSF (*n* = 1) or University of Heidelberg (*n* = 4), performing either capture-based NGS using the UCSF500 NGS Panel targeting all coding exons of 479 cancer-related genes along with select introns and upstream regulatory regions of 47 genes [12], or using a custom-designed, enrichment-based NGS panel as described previously [20], developed at the Department of Neuropathology of the University Hospital Heidelberg that targets the coding exons of 201 cancer-related genes, nine gene fusions, and selected intronic and promoter regions. Raw NGS data are available upon request.

### Statistical analyses

Samples of the *PLAGL1*- and *PLAGL2*-amplified subtypes were derived from [11] and [10]. Kruskal–Wallis test was used to assess the difference in age between the three subtypes and Dunn’s multiple comparisons test was used as a post hoc test. Survival analysis was performed using R version 4.4.2 [27]. The Kaplan–Meier method was used to determine progression-free survival (PFS) and overall survival (OS) for the *PLAG1*-fused group separately as well as in comparison with the *PLAGL1*- and *PLAGL2*-amplified subtypes. The log-rank test (p value) was used to identify differences between the Kaplan–Meier curves. Overall survival was defined as the time between first diagnosis and last follow-up date or death. PFS was defined as the time between first diagnosis and time point of first relapse or progression.

## Results

### *PLAG1* identified as a recurrent 3’ fusion partner in pediatric CNS tumors

**Fig. 1:**
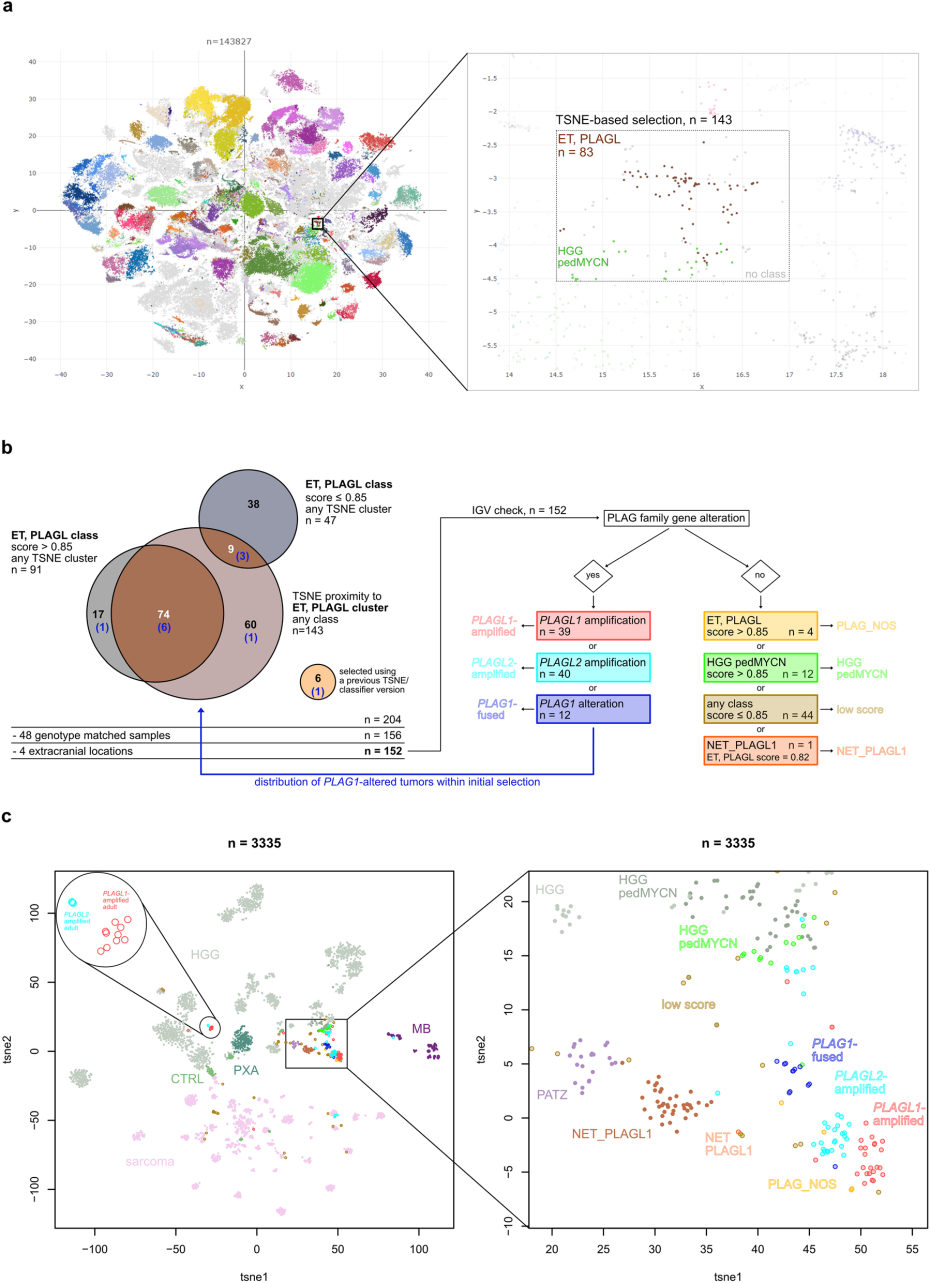
Screening of tumors either located in proximity to the ET, PLAGL methylation-based t-SNE cluster or having received a classifier score of ET, PLAGL identifies a novel epigenetically distinct subtype of ET, PLAGL characterized by *PLAG1* gene fusions. **a **Left: DNA methylation-based t-SNE analysis of >140,000 pediatric and adult tumor samples. Marked is the ET, PLAGL type. Right: enlarged depiction of the samples selected for screening and classified as ET, PLAGL, or other tumor types. Methylation classes are color-coded as described in [4]; gray color means that the sample could not be matched to any of the existing methylation classes. **b** Workflow of the screening process: samples were selected based on t-SNE (proximity to ET, PLAGL) or classifier result (score of ET, PLAGL). Methylation array-derived copy-number data were then screened in the selected samples regarding the PLAG family genes using IGV. **c** DNA methylation-based analysis using t-SNE dimensionality reduction on 91 PLAG(L)-altered tumors (*PLAGL1*-amplified, *PLAGL2*-amplified, *PLAG1*-fused) and a reference cohort of 3183 different CNS tumors including 1669 gliomas/glioneuronal tumors (HGG, adult and pediatric), 1096 sarcomas, 130 medulloblastomas, 39 supratentorial neuroepithelial tumors with *PLAGL1* fusion, and 23 neuroepithelial tumors with *PATZ1* fusion. Methylation classes are color-coded and labeled using the respective group abbreviations. ET, PLAGL tumors are differentially colored according to their altered PLAG-family gene. Samples belonging to the ET, PLAGL type are depicted enlarged on the right. Group names are: high-grade glioma (HGG), pleomorphic xanthoastrocytoma (PXA), non-tumor control samples (CTRL), sarcoma, medulloblastoma (MB), high-grade glioma MYCN subtype (HGG-pedMYCN), supratentorial neuroepithelial tumor with *PLAGL1* fusion (NET_PLAGL1), neuroepithelial tumor with *PATZ1* fusion (PATZ), and low score (samples with a score ≤0.85 for any class)

Molecular diagnostics, including DNA methylation profiling, methylation data-derived copy-number analysis, and RNA sequencing of pediatric CNS tumor samples, identified individual tumors harboring a gene fusion involving the PLAG family gene *PLAG1* as the 3’ fusion partner. Methylation profiling of those tumors [4] revealed that they were either classified as ET, PLAGL by the Heidelberg Brain Tumor Classifier (v12.8) and/or clustered in close proximity to this recently described tumor type [11] by unsupervised visualization of genome-wide DNA methylation data through t-distributed stochastic neighbor embedding (t-SNE) of > 140,000 pediatric and adult tumor samples (Figure 1a). Hence, we aimed to conduct a more detailed investigation of this elusive and potentially novel rare tumor (sub-)type and therefore screened for more tumors with *PLAG1* alterations in this set of 143,827 samples.

### Comprehensive screen identifies further tumors with *PLAG1* alteration

**Fig. 2:**
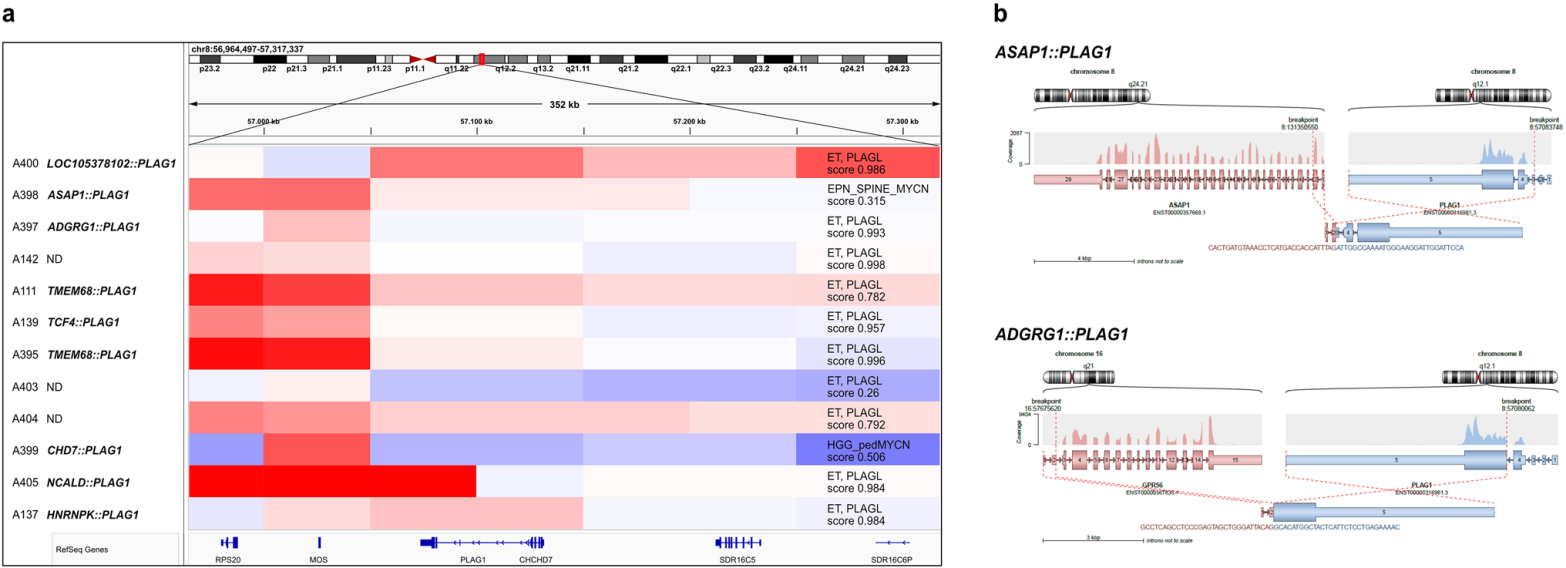
Copy-number analysis of CNS embryonal tumors with suspected *PLAG1* gene fusion. **a** Copy-number breakpoints occurring at the *PLAG1* locus on chromosome 8q12.1 indicating possible fusion events were visualized using IGV. *PLAG1* gene fusions were verified using RNA-seq, WGS, or prior institutional sequencing. Heidelberg classifier methylation class and calibrated score (v12.8) are indicated. **b** Shown are examples of the fusion caller results, including breakpoints and fusion partners

We initially selected all samples clustering close to ET, PLAGL by t-SNE analysis, independent of their classification (*n* = 143) as well as all samples whose highest classifier score was for ET, PLAGL, independent of their location on the t-SNE (*n* = 138, with an overlap of 83 samples between both; Figure 1a, b). Additionally, six samples that had been selected using a previous t-SNE and classifier version were included in our screen. After filtering out 48 duplicate samples and four samples with a known extracranial location, we analyzed the remaining 152 samples for PLAG family gene alterations by visualizing the methylation data-derived copy-number information using the Integrative Genomics Viewer (IGV) [19]. We identified 39 and 40 samples with *PLAGL1* and *PLAGL2* amplification, respectively, as well as 12 samples with a copy-number alteration within a breakpoint centered in the *PLAG1* gene locus on chromosome 8q12.1 (Fig. 1b and 2a). Seven of these twelve samples were classified as ET, PLAGL (score >0.9), while 3/12 received an ET, PLAGL score below 0.8 (0.26-0.792), and 2/12 received a low score for a different tumor type (0.315—EPN_SPINE_MYCN and 0.506—HGG_pedMYCN). All 12 of these primary intracranial tumor samples with *PLAG1* copy-number breakpoints were used for subsequent analyses. One of the remaining 61 samples was confirmed to have an *EWSR1*::*PLAGL1*-fusion and had been reported previously as supratentorial neuroepithelial tumor with *PLAGL1* fusion (NET_PLAGL1, Figure 1b) [22]. Four of the 61 samples had an ET, PLAGL score above 0.85, but no detectable alteration of either PLAG family gene, and were therefore named PLAG_NOS (not otherwise specified) (Figure 1b).

### Refined t-SNE shows *PLAG1*-altered tumors in close proximity of *PLAGL1/2*-amplified tumors

We included the information regarding PLAG family gene alteration status derived from the previous IGV screen in our subsequent t-SNE analysis where we visualized the 91 PLAG(L)-altered tumors and the 61 remaining tumors (PLAG_NOS, HGG-pedMYCN, low score, NET_PLAGL1) together with a cohort of 3183 reference tumors (total *n* = 3335) consisting of numerous different high-grade glioma (HGG, adult and pediatric, *n* = 1669), sarcoma (*n* = 1096), and embryonal tumor types (*n* = 130) as well as supratentorial neuroepithelial tumor with *PLAGL1* fusion (NET_*PLAGL1*, *n* = 39) [22] and neuroepithelial tumor with *PATZ1* fusion [2] (HGNET_PATZ, n=23) (Figure 1c). As described previously [11], the *PLAGL1*- and *PLAGL2*-amplified tumors formed separate sub-clusters within the same overarching tumor type—ET, PLAGL. In addition, the *PLAG1*-fused tumors formed a third separate sub-cluster adjacent to the two clusters with *PLAGL1/2*-amplified tumors with minimal overlap. A subset of *PLAGL1/2*-amplified tumors formed a separate cluster, which only consisted of adult patients and was not located in proximity of pediatric ET, PLAGL. Additional hierarchical clustering analysis yielded a similar grouping (supplementary Figure 5).

### Transcriptome analysis identifies multiple different 5’ fusion partners and shows similarities in gene expression with *PLAGL1/2*-amplified tumors

For 9 of the 12 *PLAG1*-altered samples, we either acquired FFPE tumor tissue to perform RNA sequencing (*n* = 6) or molecular data from the respective collaborating institution (*n* = 2) or the literature (*n* = 1) [26] to confirm a gene fusion between either exons 4 or 5 of *PLAG1* as the 3’ partner and the 5’ UTR or the initial 5’ exons of various other genes (*ASAP1*, *ADGRG1*, *TMEM68*, *NCALD*, *HNRNPK*, *CHD7*, *TCF4*, *LOC105378102*) as the 5’ fusion partner (Figure 2a, b**, **supplementary Figure 1). Breakpoints detected through our in-house analysis are displayed in Figure 2b and supplementary Figure 1, breakpoints reported to us from the collaborating institutions were (GRCh37/hg19): chr8:61,591,641 (*CHD7*)::chr8:57,083,748 (*PLAG1*), chr6:164,340,946 (*LOC105378102*)::chr8:57,080,945 (*PLAG1*), and chr18:52,899,739 (*TCF4*)::chr8:57,080,061 (*PLAG1*). Apart from the fusion events detected through transcriptome analysis, no additional recurrent oncogenic alteration was identified based on targeted DNA sequencing (*n* = 5). For example, no tumors had *SMARCB1* or *SMARCA4* alterations characteristic of ATRT, no tumors had histone H3 mutations characteristic of diffuse midline or hemispheric gliomas in children, and no tumors had *C19MC* amplification or *DICER1* mutation characteristic of embryonal tumor with multilayered rosettes (ETMR).

**Fig. 3:**
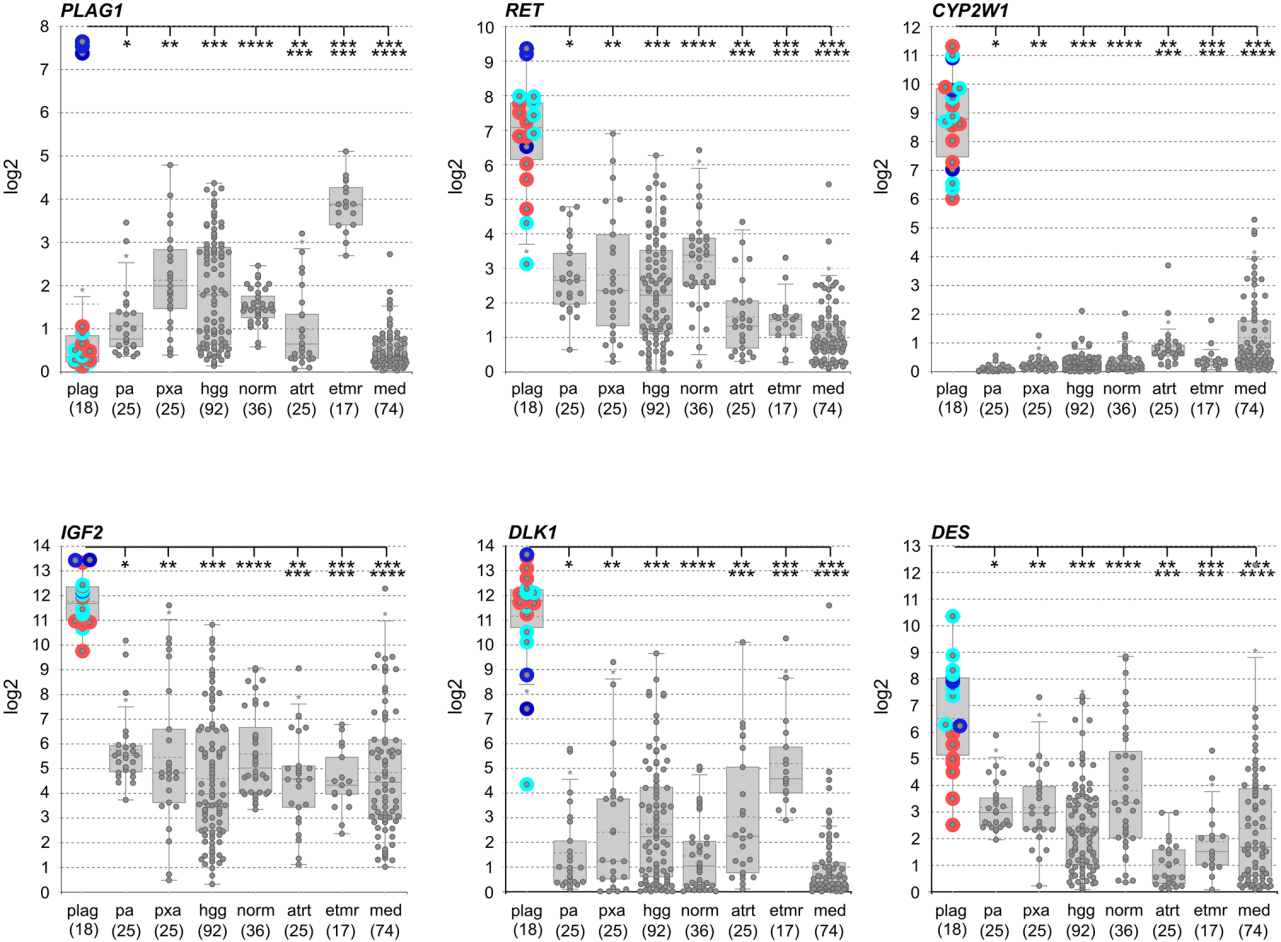
Gene expression of CNS embryonal tumors with *PLAGL1/2* amplification or *PLAG1* fusion. Boxplots comparing gene expression between CNS tumor types for a select set of genes. plag = *PLAGL1/2*-amplified and *PLAG1*-fused tumors; pa = pilocytic astrocytoma; pxa = pleomorphic xanthoastrocytoma; hgg = high-grade gliomas (G34R/V, K27M, pedRTK1/2); norm = normal brain tissues; atrt = atypical teratoid rhabdoid tumor; etmr = embryonal tumor with multilayered rosettes; med = medulloblastomas (WNT, SHH, group 3, group 4); red: samples with *PLAGL1* amplification, turquois: samples with *PLAGL2* amplification, and blue: samples with *PLAG1* fusion. Significance bars indicate groups whose differences in gene expression are statistically significant when compared to the first group (plag) (t test, Bonferroni-corrected p value = 0.00714286). *PLAG1* upregulation is statistically significant compared to all other groups when looking at *PLAG1*-fused tumors separately

In addition, we performed RNA sequencing on frozen tissue of three of the six samples to evaluate the gene expression patterns of *PLAG1*-fused tumors using the R2 Genomics Analysis and Visualization Platform (http://r2.amc.nl). *PLAG1*-fused tumors showed upregulation of *PLAG1* (Figure 3), which is downregulated postnatally in normal brain and cerebellum (supplementary Figure 2b) (https://apps.kaessmannlab.org/evodevoapp/) [5]. In addition, prenatal expression levels of *PLAG1* are lower in cerebral and cerebellar tissues than those of *PLAGL1* or *PLAGL2*. Comparative gene expression analysis using a reference cohort of 294 samples from other CNS tumor and normal tissues [HGGs with H3 G34R/V or K27M mutation and GBM_pedRTK1 or 2 (*n* = 92), PA with *BRAF* fusion (*n* = 25), PXA (*n* = 25), normal brain tissue (*n* = 36), embryonal tumors such as ATRT, ETMR, or medulloblastomas (*n* = 116)] showed upregulation of ET, PLAGL-associated genes, such as *CYP2W1*, *RET*, *IGF2*, *DLK1*, and *Desmin*. Desmin expression was more elevated in *PLAG1*-fused tumors compared to *PLAGL1*-amplified tumors, and similar to *PLAGL2*-amplified tumors.

For a better understanding of the similarities and differences of all three PLAG gene family members, especially with regards to the here described gene fusions, but also the differences in clinical behavior, some of the structural differences and similarities described in the literature are summarized in supplementary Figure 2a with a special focus on the domains that are preserved by the detected *PLAG1* fusions. The fusion breakpoints lead to preservation of either the full translated region (exons four and five) or the majority of the translated region (exon five only) of *PLAG1*. This leads to overexpression of the full or partly truncated wild-type gene preserving either all zinc fingers or zinc fingers 3-7 of the DNA-binding domain, respectively.

### Histopathological characteristics of *PLAG1*-altered CNS tumors

**Fig. 4:**
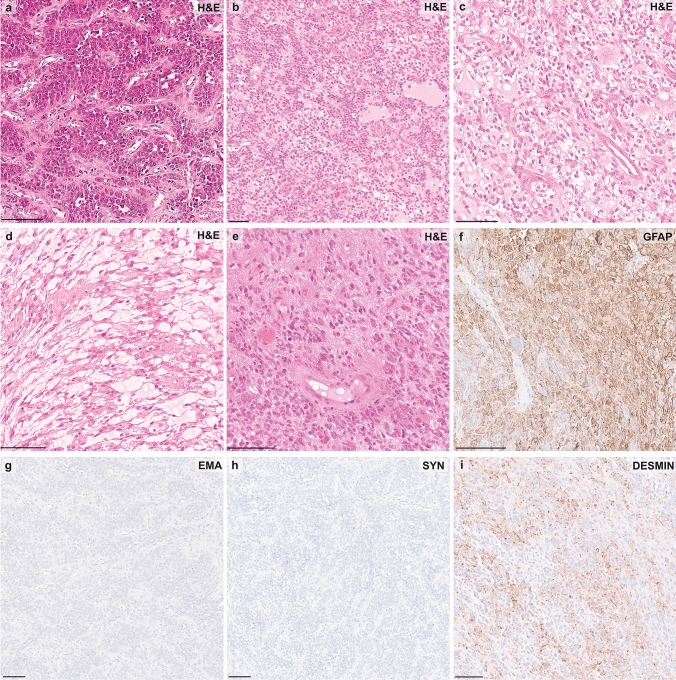
Histologic and immunohistochemical features of CNS embryonal tumors with *PLAG1* fusion. High-resolution H&E-stained histology images of *PLAG1*-fused tumors. **a, b** Focally, the tumors exhibit features reminiscent of embryonal morphology, including densely cellular areas composed of primitive small round blue cells. **b** A subset of tumors contains follicle-like structures with homogeneous eosinophilic secretions. **c, d** Other regions demonstrate divergent differentiation, including clear cell changes and microcystic architecture. **a, e** An ependymoma-like appearance with perivascular pseudorosettes is also noted. **f** Immunohistochemistry reveals diffuse GFAP positivity. **g, h** EMA and synaptophysin are negative. **i** Desmin shows focal cytoplasmic positivity

A histopathological review of a subset of *PLAG1*-fused tumors (*n* = 4) revealed marked morphologic heterogeneity. While all tumors were predominantly densely cellular and composed in part of primitive small round blue cells (Figure 4a, b), their overall appearance varied considerably. Three tumors exhibited an ependymoma-like pattern with perivascular pseudorosettes (Figure 4a), and two showed follicle-like structures with homogeneous eosinophilic secretions (Figure 4b). Clear cell differentiation and microcystic changes were noted in two cases (Figure 4c, d). Focal areas of pronounced nuclear pleomorphism, characterized by variation in nuclear size, shape, and chromatin distribution, were observed (Figure 4e). Additional features included calcifications in one tumor and necrosis in another. The vasculature consisted of thin-walled or hyalinized blood vessels, without evidence of glomeruloid microvascular proliferation. Immunohistochemical analysis demonstrated diffuse GFAP positivity in all tumors (Figure 4f), S100 expression in two cases, and consistent negativity for OLIG2, EMA (Figure 4g), and synaptophysin (Figure 4h). CD99 was positive in one tumor, while LIN28A was absent in all. Desmin expression was observed only focally. The Ki-67 labeling index ranged from 10% to 20%.

### Patient characteristics and survival resembled *PLAGL1/2*-amplified tumors

**Fig. 5:**
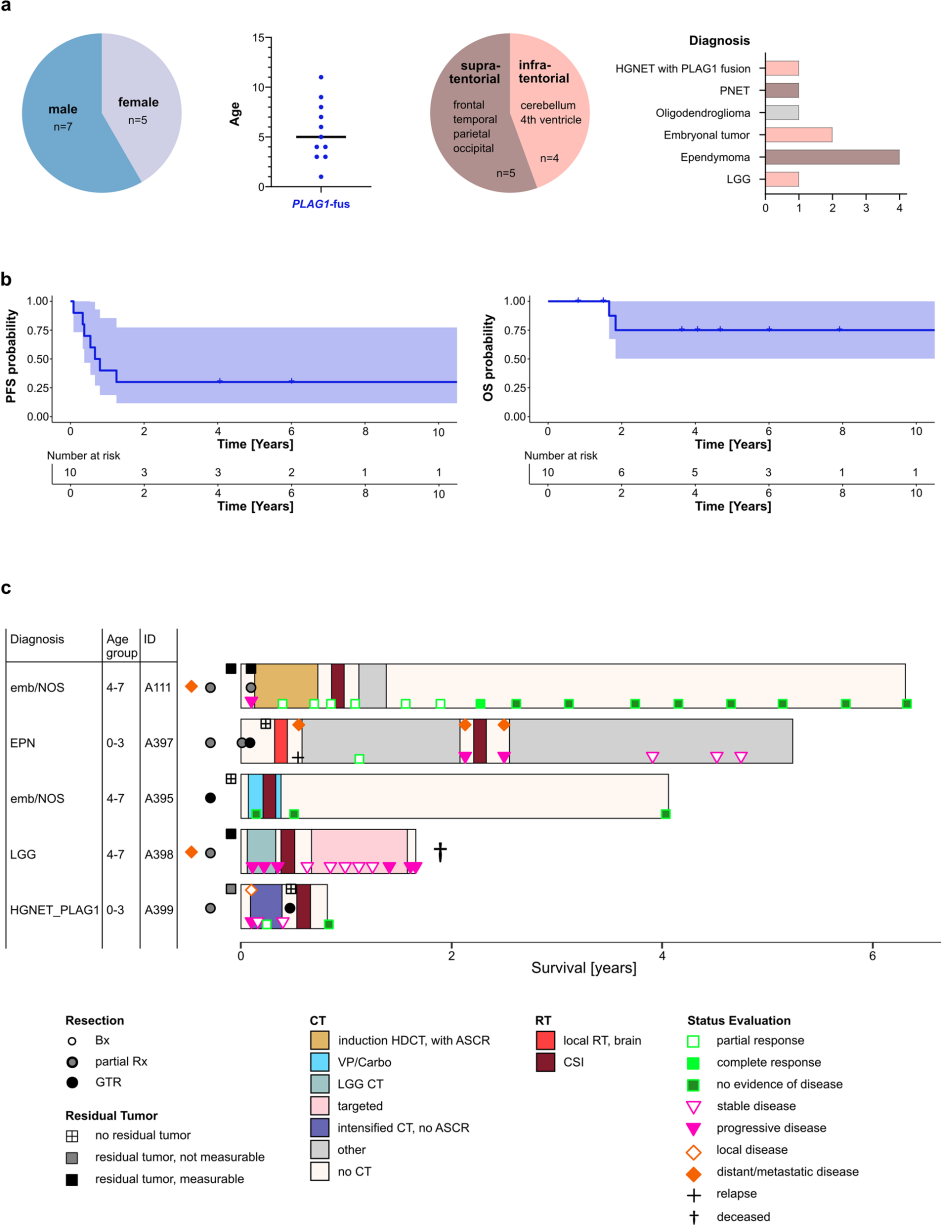
Patient characteristics and clinical outcomes of patients with *PLAG1*-fused CNS embryonal tumors. **a** Summary of sex, age, localization of tumor, and primary diagnosis. **b** Kaplan–Meier plots showing progression-free survival (PFS) and overall survival (OS). **c** Swimmer plot showing a detailed summary of clinical information and outcome for five patients. OS is shown on the x-axis. IDs, age, and primary diagnosis are listed for each patient on the left. Bars are colored according to treatment (CT and RT). Extent of resection, residual tumor amount after resection, and response to treatment were determined through review of MRI scans and displayed through the different symbols. Death of a patient is symbolized by an obelisk behind the respective bar. Abbreviations: emb, embryonal; NOS, not otherwise specified; EPN, ependymoma; LGG, low-grade glioma; HGNET_PLAG1, high-grade neuroepithelial tumor with *PLAG1* fusion; Bx, biopsy; Rx, resection; GTR, gross total resection; CT, chemotherapy; HDCT, high-dose chemotherapy; ASCR, autologous stem cell rescue; VP, Vepesid; Carbo, Carboplatin; RT, radiotherapy; CSI, craniospinal irradiation

The available patient characteristics are visualized in Fig. 5 and supplementary Figure 3 and summarized in supplementary Table S1. Out of the 12 patients with *PLAG1*-fused tumors, 7 patients were male, and 5 were female. Five tumors were localized in the supratentorial (frontal, temporal, parietal, and occipital lobes), and four were localized in the infratentorial region (cerebellum, fourth ventricle). The original histopathologic diagnoses comprised low-grade glioma (LGG) (*n* = 1), embryonal tumor NOS (*n* = 2), high-grade neuroepithelial tumor (HGNET) with *PLAG1* fusion (*n* = 1), primitive neuroectodermal tumor (PNET) (*n* = 1), supratentorial ependymoma (*n* = 4), and oligodendroglioma (*n* = 1). The age at initial diagnosis was available for 11 patients and ranged between 1 and 11 years with a median of 5 years (Figure 5a). No significant difference in age at diagnosis was found between *PLAG1*-fused and *PLAGL1-*amplified or *PLAGL2*-amplified tumors (post hoc Dunn’s test, *p* = 0.7906 and 0.1198, respectively). As reported previously, *PLAGL1*- and *PLAGL2*-amplified tumors differed significantly regarding their age at diagnosis, which could be confirmed in the current cohort where we combined all available *PLAGL1/2* cases [age at diagnosis available for *n* = 10 (*PLAGL1*-amplified) and *n* = 15 (*PLAGL2*-amplified) patients, respectively] (Kruskal–Wallis test, *p* = 0.0044, post hoc Dunn’s test, *p* = 0.0042) [10, 11] (supplementary Figure 3b). Clinical outcome data were available for 10 patients with *PLAG1*-fused tumors and for 11/13 patients with *PLAGL1/2*-amplified tumors, respectively, from previously published cohorts [10, 11]. Five-year PFS and OS for patients with *PLAG1*-fused tumors were 30.0% and 75.0%, respectively (Figure 5b). For patients with *PLAGL1*-amplified tumors, PFS/OS was 90.0%/75.8% and for patients with *PLAGL2*-amplified tumors PFS/OS was 34.6%/32.0% (supplementary Figure 3a).

### MR imaging features

MRI data were available for five patients. In four cases, the tumor was located in the posterior fossa, while in the remaining case, it was in the left parietal lobe. Two of the posterior fossa tumors were already disseminated at diagnosis, with one also extending into the spinal canal. The parietal tumor developed leptomeningeal supratentorial metastases 6 months after diagnosis and later showed spinal metastases during follow-up. The other two posterior fossa tumors remained localized throughout the study period. Locations of the primary tumors and distant relapses are summarized in supplementary Tables S3 and S4.

The MRI characteristics of these tumors were diverse, with no consistent pattern observed (supplementary Figure 4). They appeared as either solid or mixed solid-cystic masses and exhibited varying degrees of contrast enhancement, ranging from none to strong. On T2-weighted imaging, they were mostly hyper- and/or isointense. Diffusion-weighted imaging was available for four cases, of which two showed diffusion restriction. Tumor volumes at diagnosis ranged from 15 to 183 mL. Hemorrhage and/or calcification were present in three cases.

### Treatment regimens were heterogeneous, with some favorable responses to chemotherapy and radiotherapy

Applied treatment regimens varied widely depending on initial diagnosis and patient age (Fig. 5c, supplementary Tables S2 and S5). All but one patient received chemotherapy as part of their primary adjuvant treatment—high-dose chemotherapy with autologous stem cell rescue (HDCT/ASCR, *n* = 1), intensified induction chemotherapy (*n* = 1), carboplatin/etoposide combination (*n* = 1), or low-grade glioma (LGG) tailored chemotherapy (*n* = 1). Favorable responses to chemotherapy alone were achieved in two patients in the primary setting upon HDCT/ASCR and intensified induction CT and in the relapse setting upon temozolomide (TMZ). One patient showed tumor progression while receiving LGG tailored chemotherapy. One patient with initial diagnosis of an ependymoma was not treated with chemotherapy within the adjuvant post-surgical setting. Four patients with detailed treatment information received adjuvant radiotherapy (RT) following their initial resection—three patients received craniospinal irradiation (CSI/boost) and one patient received local RT to the tumor bed. Two patients initially presented with disseminated distant disease. After partial resection, one of them experienced tumor progression prior to initiation of adjuvant therapy and another one progressed during LGG tailored chemotherapy. Another patient with localized disease developed progressive disease after partial resection prior to the start of adjuvant therapy. Of the five patients, three are in remission (at 72, 49, and 10 months after initial presentation), all after combined CSI/boost and chemotherapy, while one patient is alive with stable disease after several lines of treatment (at 56 months after initial presentation), and one patient has succumbed to his disease (at 20 months after initial presentation).

## Discussion

We describe a rare novel subtype of pediatric CNS embryonal tumor, which is defined by a specific DNA methylation signature and gene fusion of *PLAG1* as the 3’ partner and various other genes as 5’ partners, leading to upregulation of *PLAG1*. The location of the fusion involving the regulatory regions of the 5’ partners as well as the untranslated region (UTR) before exon 4 of *PLAG1* suggests promoter hijacking as the mechanism leading to upregulation of *PLAG1* expression in those tumors, similar to what is reported for pleomorphic adenomas of the salivary gland [9]. In both scenarios, upregulation of either the full or a truncated form of *PLAG1*, the specific DNA-binding domains, i.e., zinc finger three, which interacts with the G-cluster, and zinc fingers six and seven, which bind the core motif, are preserved [31, 33], implying that the functionality of *PLAG1* is likely preserved as well. This hypothesis is supported by the observed transcriptional upregulation of known *PLAG1* targets, such as *IGF2*, in *PLAG1*-fused tumors [6].

Four samples were available for histological review and showed heterogeneous morphology. While the number of samples with IHC analysis is too small to draw general conclusions, the available results point toward a slightly different morphology compared to *PLAGL1/2*-amplified samples with some overlapping embryonal features such as primitive small round blue cells, but at the same time an ependymoma-like appearance with perivascular pseudorosettes in three of four cases as well as GFAP positivity in all four investigated samples, both features that were not or only rarely observed in *PLAGL1/2*-amplified tumors [11, 25]. Despite this divergence, we still consider characterization as embryonal tumor by histology (small round blue cells), by methylation analysis (proximity to the *PLAGL1/2*-amplified tumors), and by outcome (early progressions or relapses in four out of the five cases with complete clinical data and imaging) as the best fitting classification.

We further compare gene expression of *PLAG1*-fused tumors and *PLAGL1/2*-amplified tumors by RNA sequencing. Similar to the *PLAGL1/2*-amplified tumors, *PLAG1*-fused tumors show upregulation of the respective altered PLAG family gene as well as confirmed *PLAGL1/2* downstream targets such as the above-mentioned *IGF2*, but also *DLK1*, *RET*, *CYP2W1*, and *Desmin* [6, 11, 32]. Overexpression of this specific gene set seems to be uniquely characteristic of *PLAGL1/2*-amplified tumors, as we do not find this combination, and especially not *CYP2W1* expression, in any of the other investigated brain tumor types. The fact that the identical gene set also shows high expression in *PLAG1*-fused tumors, supports the classification of these three subtypes into a single overarching tumor entity. Similar Desmin expression in *PLAG1*-fused and *PLAGL2*-amplified tumors suggests further potential biological similarity between these two subtypes as is described in general in the literature: Differences and similarities have been described for the three PLAG family genes, *PLAG1*, *PLAGL1*, and *PLAGL2*, which are all transcription factors. While *PLAG1* and *PLAGL2* have been described as proto-oncogenes, *PLAGL1* seems to act in a context dependent manner either as a tumor suppressor gene or, as recently described by us and others, as a proto-oncogene in the brain tumor context [1, 7, 11, 22].

Analysis of the expression pattern of all three PLAG family genes in brain and cerebellum throughout human development shows upregulation during prenatal development and downregulation postnatally. While the pattern is similar for *PLAGL1* and *PLAGL2*, it seems that there is even tighter regulation for *PLAG1*, which is only expressed during the first trimester (https://apps.kaessmannlab.org/evodevoapp/) [5]. This highlights developmental differences between the three PLAG family genes in general, and indicates functional consequences in tumors with upregulation of the normally suppressed or weakly expressed PLAG family genes as well as dysregulation of their targets. Further differences in the primary sequence exist in the transactivation domains, which differ between all three PLAG family genes, but show more similarity between *PLAG1* and *PLAGL2* compared to *PLAGL1* in terms of their capacity to activate gene transcription [8]. Differences regarding DNA binding have also been reported, as all three PLAG proteins bind different DNA sequences in a distinct fashion, despite their sequence homology in the DNA-binding domain—PLAGL1 does not bind the G-cluster as do PLAG1 and PLAGL2 [6].

By t-SNE dimensionality reduction of DNA methylation profiles, *PLAG1*-fused tumors show a methylation pattern closely related to the one characteristic of *PLAGL1*- and *PLAGL2*-amplified tumors [11], but otherwise epigenetically distinct from all other described CNS tumor types. The close proximity in our t-SNE analysis and overall epigenetic and transcriptional similarity points to *PLAG1*-altered tumors as a third subtype of ET, PLAG(L). Seven of the twelve *PLAG1*-fused samples received a Heidelberg classifier (v12.8) calibrated score above 0.9 for CNS embryonal tumors with PLAGL amplification (ET, PLAGL) indicating their belonging to that tumor type. Three of 12 samples received a score between 0.26 and 0.792, while 2/12 samples received low scores for different classes—0.506 (HGG_pedMYCN) and 0.315 (EPN_SPINE_MYCN)—even though they clustered close to the ET, PLAGL group in our t-SNE analysis. We could confirm *PLAG1*-fusions in these two samples by RNA-seq with concurrent absence of *MYCN* amplifications in the methylation derived copy-number data (data not shown). The reason for the difference in classifier scores for *PLAG1*-fused tumors remains to be investigated, but inclusion of this tumor group in future iterations of the reference database will hopefully help their prospective accurate identification. It is important to note that for the time being using the current version of the Heidelberg classifier, one needs to consider a diagnosis of *PLAG1*-fused tumor even when a low calibrated score for ET, PLAGL, or a different group is the methylation classifier output, at least in cases showing a concurrent proximity to ET, PLAGL in the t-SNE analysis. When in doubt, additional analyses such as RNA sequencing to confirm *PLAG1* fusions as well as overexpression of *PLAG1* and *PLAG1* targets could help to secure accurate diagnosis in such cases.

Similar to what we reported for *PLAGL1/2*-amplified tumors [11], initial diagnoses and tumor localizations varied for patients with *PLAG1*-fused tumors and included different high- and low-grade tumor entities as well as different infratentorial and supratentorial locations, leading to different treatment regimens. Median age was 5.0 years with no significant difference relative to *PLAGL1-* or *PLAGL2*-amplified tumors (median age 10.5 and 2.0 years, respectively). Progression-free survival was low for *PLAG1*-fused and *PLAGL2*-amplified cases, and the observed clinical behavior was comparable. In the cohort of five patients with complete clinical data, three patients presented with metastases at diagnosis (*n* = 2) or at relapse (*n* = 1). Early relapses or progressions occurred before initiation of adjuvant therapy (*n* = 2), during low-grade glioma tailored chemotherapy (*n* = 1), and after gross total resection and local irradiation (*n* = 1). In contrast, all three patients that received combined treatment with CSI/boost and chemotherapy remained free of progression within the observation period. Notably, the number of cases is very low and the risk of overinterpretation is therefore significant. However, there are only two other cases reported so far; both patients were also treated with CSI/boost [26] (one of these corresponds to our case A139). Given the embryonal-like pathological presentation and gene expression profile and the observed appearance of metastases, this treatment approach may be justified, while robust conclusions cannot be drawn from our series. As discussed for the *PLAGL1/2*-amplified tumors [10], the classification of *PLAG1*-fused tumors as an embryonal tumor should not automatically imply a specific treatment regimen. The question if treatment regimens used for other embryonal tumors as intensified chemotherapy or CSI are the most beneficial treatment approaches in the case of a *PLAG1*-fused tumor remains to be clarified. Likewise, our series cannot answer the question, if younger children not amenable for treatment with CSI may benefit from local radiotherapy when combined with chemotherapy treatment. Due to the rarity of the disease and given the lack of a standard treatment approach, data on treatment and outcome should optimally be collected for all patients. Only by broad collaboration and pooling of data can more solid evidence be generated to meaningfully inform future clinical trials.

In our previous publication on ET, PLAGL, we had reported three samples without apparent PLAG family gene alteration, specifically without amplification, that we called *PLAGL1*-like (*n* = 2) and *PLAGL2*-like (n = 1) based on their location of the t-SNE [11]. Re-examining the methylation derived copy-number data for all three as well as the fusion caller results after RNA sequencing for one sample (data not shown), we are unable to identify any PLAG family gene alterations, including *PLAG1* fusions, indicating an additional different molecular event driving these tumors to become PLAG(L)-like based on epigenetic profile.

Of note, CNS embryonal tumors with alteration of one of the PLAG family genes should be clearly differentiated from the supratentorial neuroepithelial tumors with *PLAGL1* fusion (NET_PLAGL1), which can be epigenetically distinguished from ET, PLAGL and which were described as being more ependymoma-like, and whose clinical behavior was not yet reported [22].

**Fig. 6:**
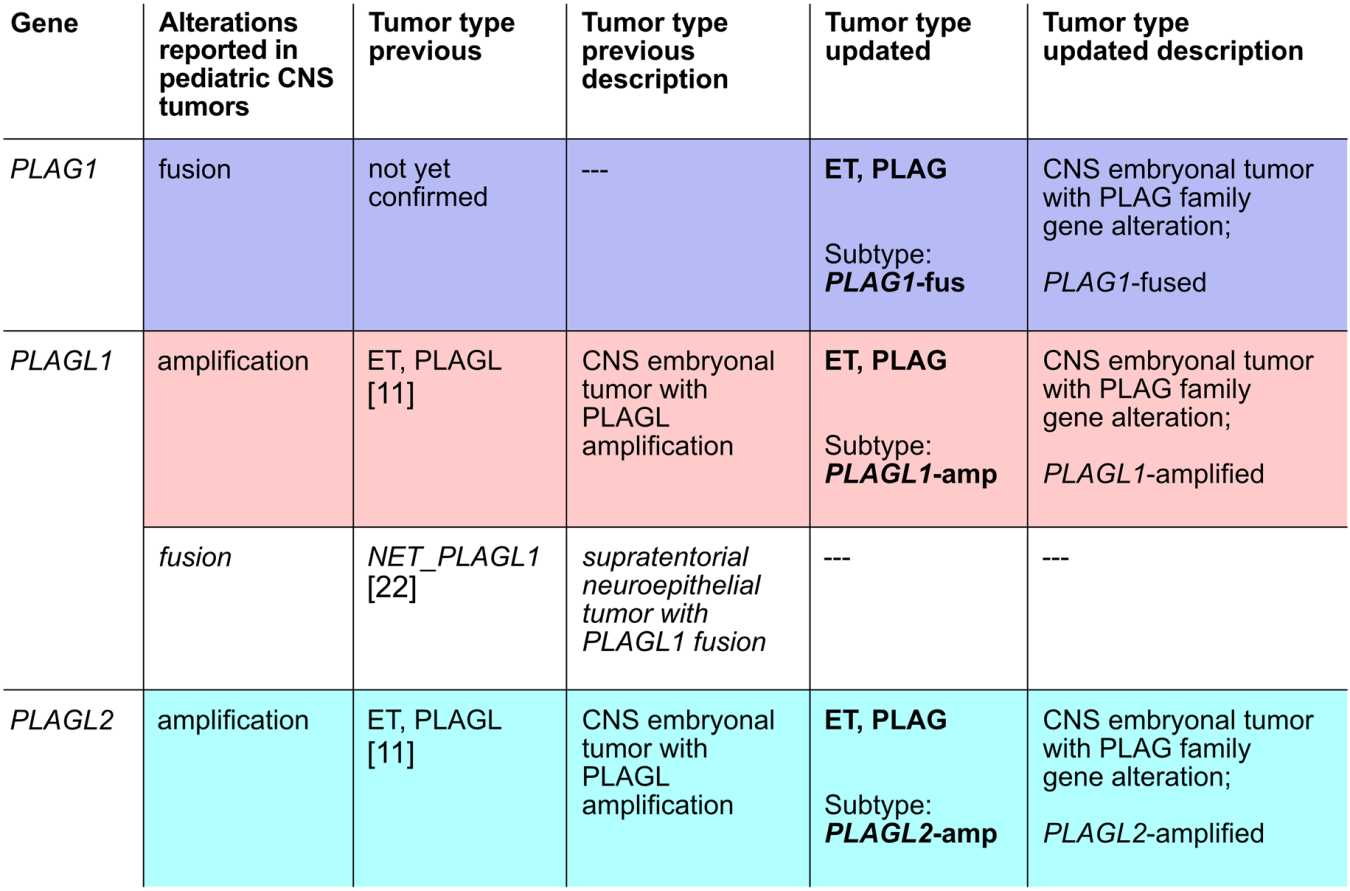
Overview of an updated nomenclature of CNS tumors with PLAG family gene alteration including specification of the respective subtype

Overall, these data support the classification of *PLAG1*-fused tumors as a distinct third subtype of ET, PLAGL alongside the previously characterized *PLAGL1*- and *PLAGL2*-amplified subtypes. To account for all three subtypes in the nomenclature, we propose to rename ET, PLAGL to ET, PLAG—CNS embryonal tumor with PLAG family gene alteration—and, especially with regards to the differences in survival and between the recommended treatments [10], advocate for an additional specification of the respective subtype, i.e. *PLAG1*-fused, *PLAGL1*-amplified, or *PLAGL2*-amplified as proposed in Fig. 6.

## Conclusion

We describe a novel subtype of CNS embryonal tumor with *PLAG1* gene fusion that is driven by upregulation and subsequent overexpression of wild-type *PLAG1* through promoter hijacking. This subtype is epigenetically distinct from all other known brain tumor subtypes, but biologically related to the previously described CNS embryonal tumor with PLAGL amplification. Like *PLAGL1*- and *PLAGL2*-amplified tumors, *PLAG1*-fused tumors show high expression of typical PLAG family downstream genes, dysregulation of imprinted genes, and overexpression of the candidate drug targets *RET* and *CYP2W1*. We therefore propose to rename the previous CNS embryonal tumor with PLAGL amplification (ET, PLAGL), which only includes *PLAGL1/2*-amplified tumors, to CNS embryonal tumor with PLAG family gene alteration (ET, PLAG) with a further specification of the three subtypes *PLAGL1*-amplified, *PLAGL2*-amplified, and *PLAG1*-fused. Preliminary analyses of the clinical behavior of *PLAG1*-fused tumors point to a certain similarity with *PLAGL2*-amplified tumors, but this remains to be investigated in a larger cohort as we cannot draw reliable conclusions based on the currently limited sample size.

## Supplementary Information

Below is the link to the electronic supplementary material.

Supplementary file1 (PDF 819 KB)

Supplementary file2 (XLSX 24 KB)

## Data Availability

DNA methylation data (IDAT files) of the 12 *PLAG1*-fused cases are accessible through The German Human Genome-Phenome Archive (GHGA accession # GHGAS82289365639281). Raw NGS data are available upon request.
