# Supplementary material for: PLAG1 fusions define a third subtype of CNS embryonal tumor with PLAG family gene alteration
Source: Acta Neuropathol. 2025 Aug 2;150(1):12. doi: 10.1007/s00401-025-02917-z (PMC12317869; doi:10.1007/s00401-025-02917-z)
Supplement: Supplementary file 1 — Supplementary file1 (PDF 819 KB) [file 401_2025_2917_MOESM1_ESM.pdf]

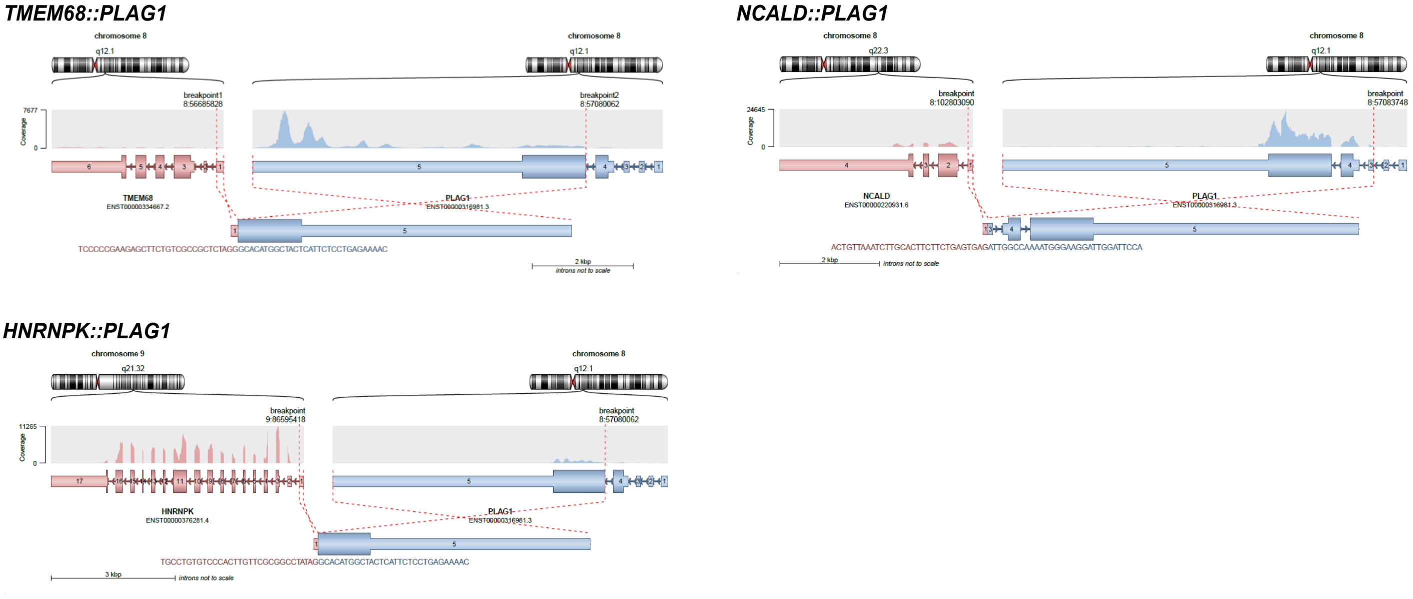

Supplementary Figure 1. Results of the fusion caller including breakpoints and fusion partners.

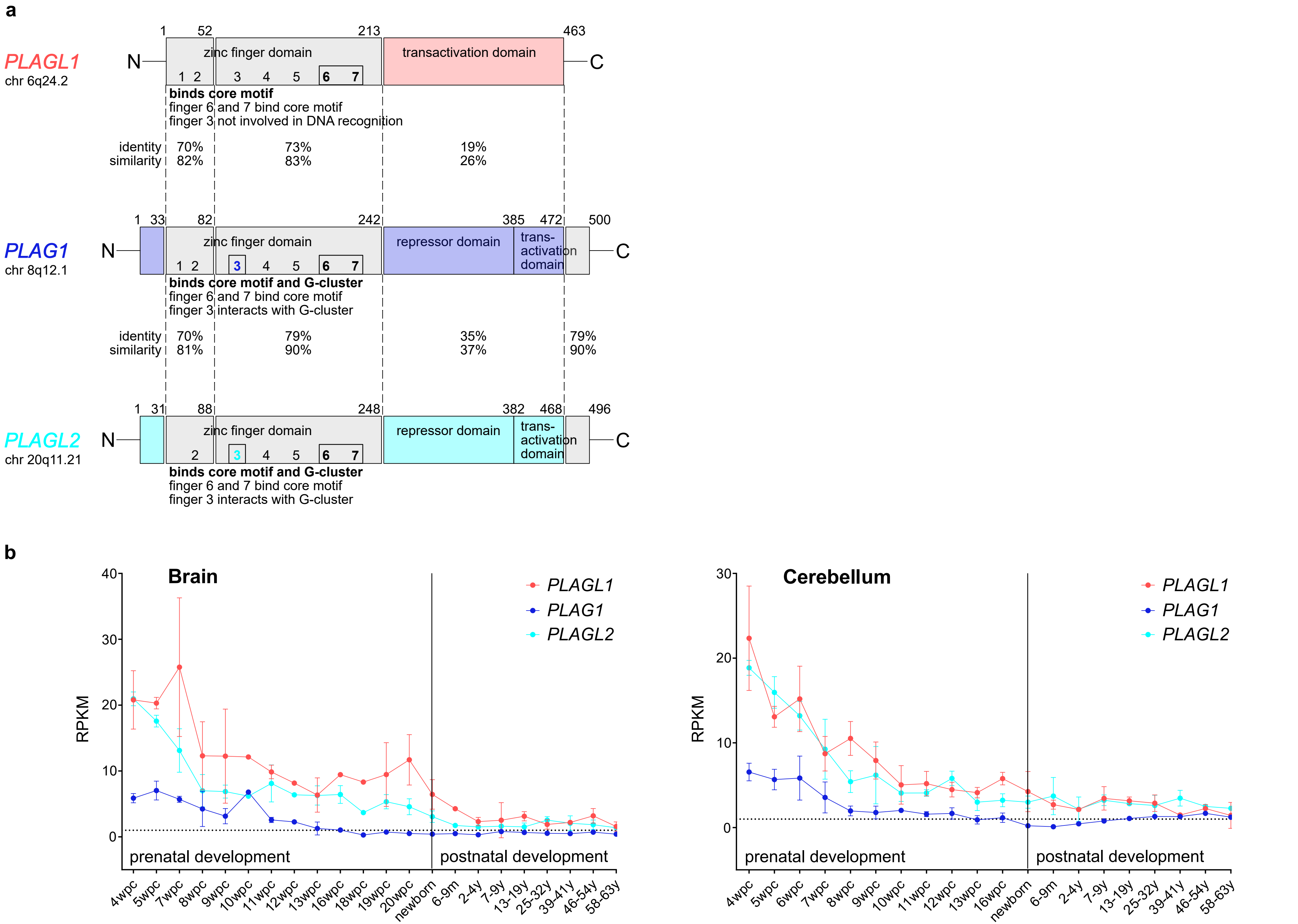

Supplementary Figure 2. Differences and similarities of the three PLAG family genes. **a** Structural comparison of *PLAG1*, *PLAGL1*, *PLAGL2*. Differences in the respective domains are indicated by color, similarities are displayed in grey. Figure was adapted from Kas et al., Hensen et al., and van Dyck et al. [6, 8, 31]. **b** Comparison of expression levels of the three PLAG family genes during development using RPKM values extracted from the evo-devo app (https://apps.kaessmannlab.org/evodevoapp/) [5].

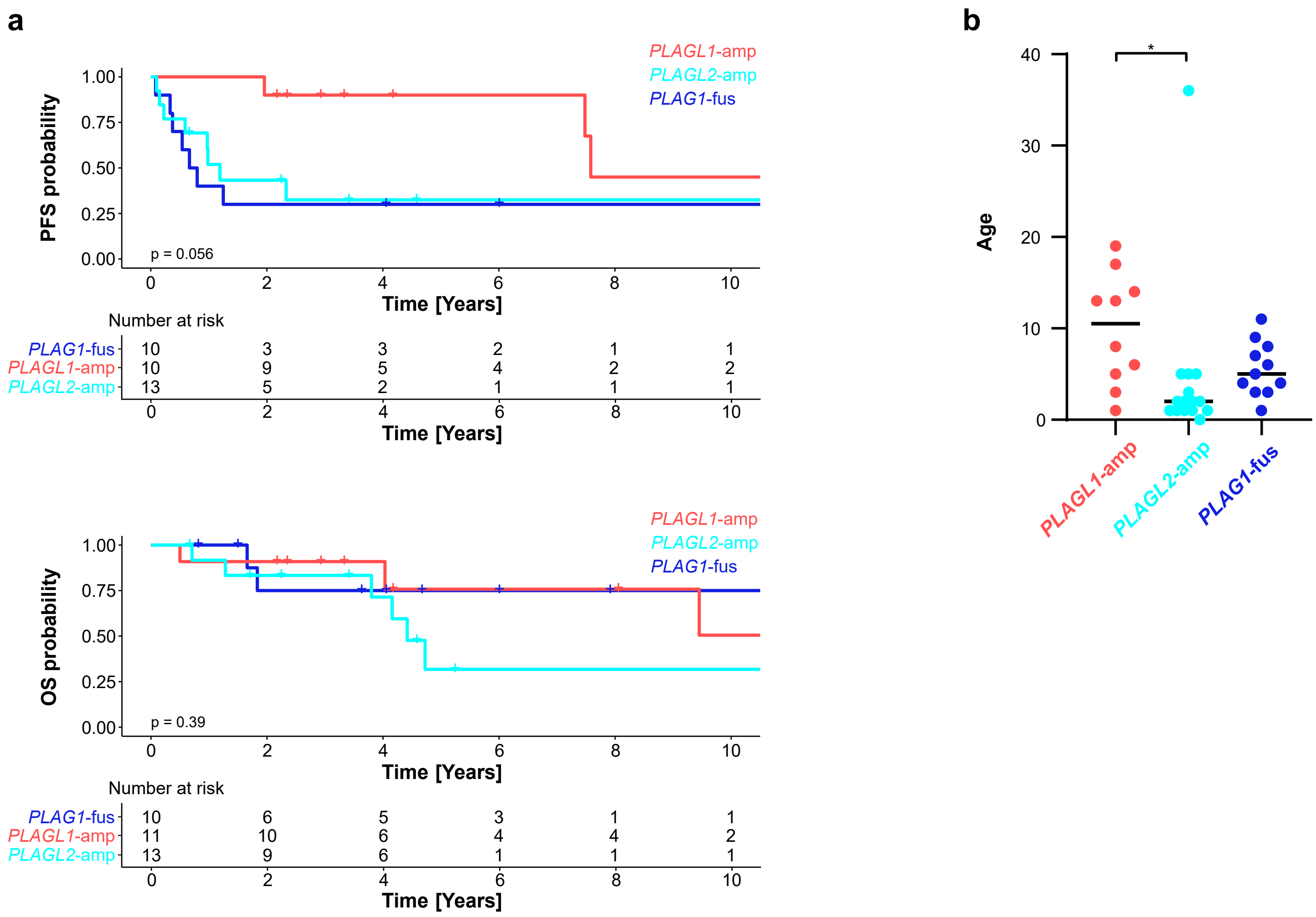

Supplementary Figure 3. Comparison of outcome and age in patients with PLAG family gene altered (*PLAG1*-fused, *PLAGL1*-amplified, *PLAGL2*-amplified) CNS embryonal tumors. **a** Kaplan-Meier plots showing progression-free survival (PFS) and overall survival (OS). **b** Age distribution and median age per group. Significant differences between groups are indicated by an asterisk.

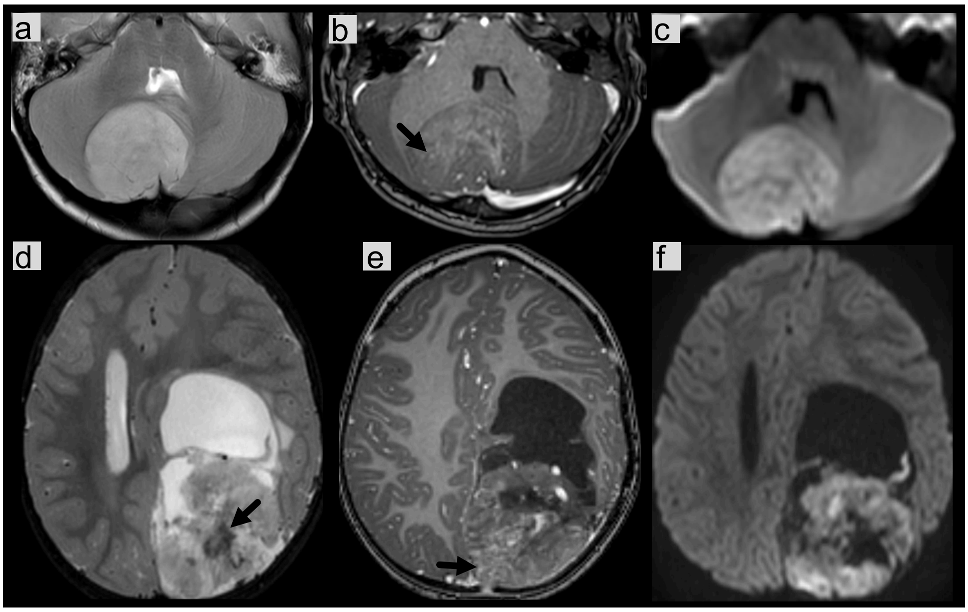

Supplementary Figure 4. Selected MR images of two patients at diagnosis: **a** posterior fossa tumor in a 5-year-old girl (**a-c**) appears well-demarcated with iso- to hyperintense signal on T2-weighted imaging (**a**), minimal contrast enhancement (arrow in **b**), and diffusion restriction on the B1000 image (**c**), with corresponding decreased ADC values (not shown). A left parietal tumor of a 3-year-old boy (**d-f**) demonstrates both solid and cystic components. It shows heterogeneous T2 signal intensity with hyper-, iso-, and hypointensity (**d**) and areas suggestive of hemorrhage and/or calcifications (arrow in **d**, confirmed on SWI; not shown). The tumor shows heterogeneous, mild contrast enhancement (arrow in **e**) and diffusion restriction on the B1000 image (**f**), with decreased ADC values (not shown).

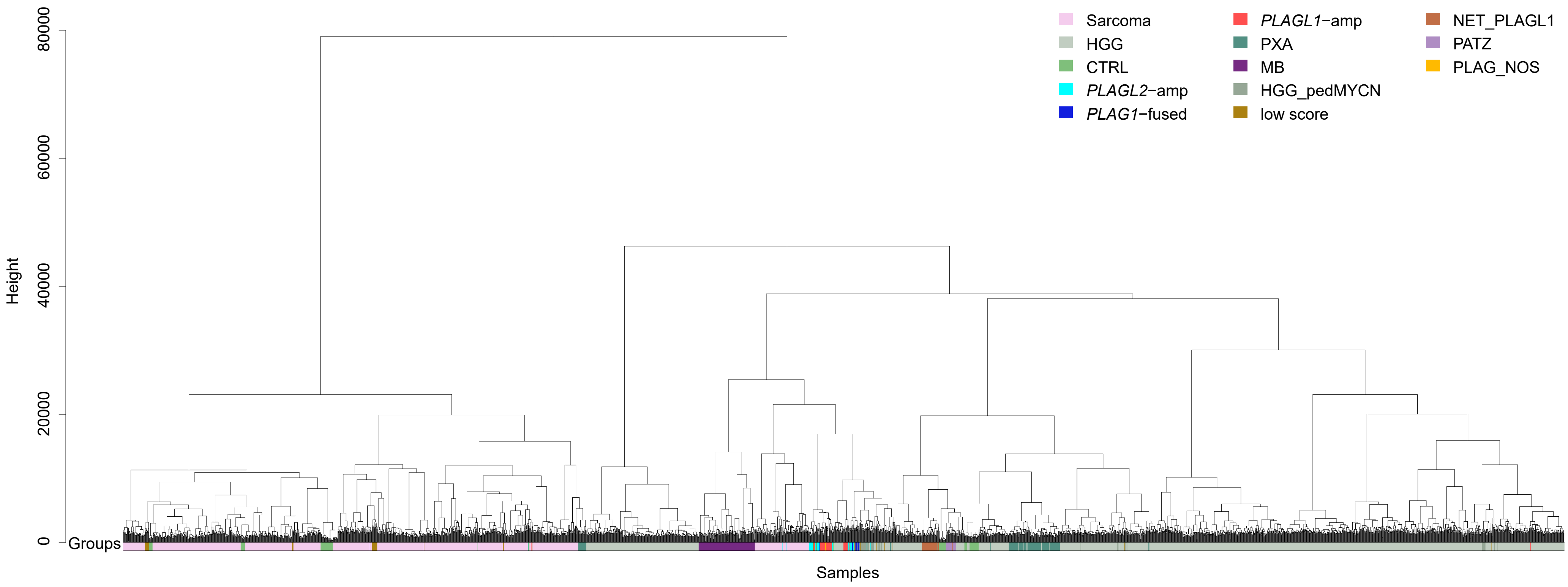

Supplementary Figure 5. Hierarchical clustering dendrogram of samples. Dendrogram showing hierarchical clustering of 3,335 samples based on the 10,000 most variable CpG sites using Manhattan distance and Ward.D2 clustering method. Samples are colored with the same color scheme as used in the t-SNE visualization in Figure 1c. The dendrogram reveals distinct clustering patterns that correspond to different tumor types, consistent with the groupings observed in t-SNE analysis. Height on the y-axis represents the distance at which clusters are merged, with higher values indicating greater dissimilarity between clusters.
